# From a single *C*-mannose to multiple *C*-mannosyltransferases

**DOI:** 10.1016/j.jbc.2026.111266

**Published:** 2026-02-06

**Authors:** Hans Bakker, Christoph Garbers, Françoise H. Routier

**Affiliations:** Institute of Clinical Biochemistry, Hannover Medical School, Hannover, Germany

**Keywords:** *C*-mannose, *C*-mannosyltransferases, post-translational modification, cytokine receptor, thrombospondin type 1 repeats

## Abstract

Glycosylation is an evolutionarily conserved post-translational modification of most proteins that are either secreted from cells or remain embedded within membranes as transmembrane proteins. It controls protein stability, plasma half-life, and intracellular trafficking and can contribute to the actual biological function of the protein. Protein glycosylation can be divided into *N*-linked glycosylation, which refers to the linkage of an oligosaccharide to the amide nitrogen of an asparagine residue; *O*-glycosylation, which describes attachment of an oligosaccharide to the hydroxyl oxygen of a serine residue or a threonine residue; and *C*-mannosylation, a rare modification in which a mannose residue is bound to the indole of a tryptophan residue *via* a carbon–carbon linkage. In this review, we summarize current knowledge about *C*-mannosylation. We describe how *C*-mannosylation was initially discovered and on which types of proteins it usually occurs. We explain the operation of the *C*-mannosyltransferases, the enzymes that attach the mannose to the substrate proteins, and which conformations the *C*-mannose adopts. Furthermore, we summarize what is known so far about the influence of the *C*-mannosylation on the function of the actual protein. Our review highlights an often overlooked post-translational modification as an important regulator of protein function.

The majority of proteins that travel through the eukaryotic secretory pathway become modified by various glycans. Three main types of protein glycosylation can be distinguished based on the linkage to the protein (1). *N*-glycosylation is highly conserved in eukaryotes and involves the transfer of an oligosaccharide from a lipid-linked donor to an asparagine residue. *O*-glycans comprise diverse structures, both with respect to the acceptor amino acid and the transferred sugar residue, and are initiated with the transfer of a monosaccharide to the protein that can be subsequently extended. Tryptophan (W) *C*-mannosylation is the only known type of protein *C*-glycosylation in higher eukaryotes (Fig. 1).

**Figure 1:**
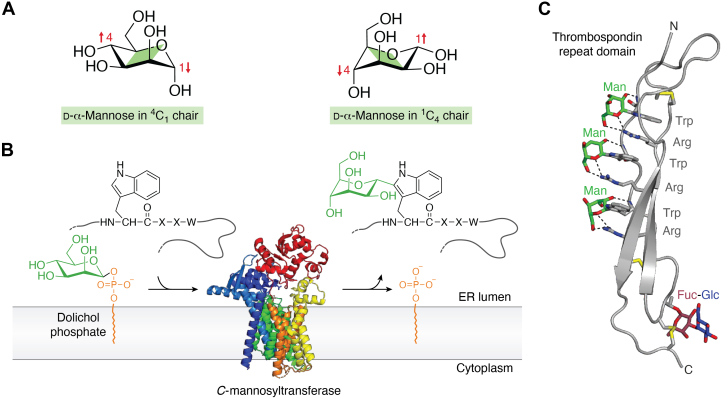
**Protein C-mannosylation**. *A*, ^4^C_1_ and ^1^C_4_ chair conformations of α-mannose. *B*, *C*-mannosyltransferases use dolichol-phosphate mannose as a donor substrate and transfer α-mannose to the WxxW/C consensus sequence in unfolded proteins on the luminal side of the endoplasmic reticulum. When bound to tryptophan, mannose preferably adopts the ^1^C_4_ conformation. The panel displays *Caenorhabditis elegans* DPY-19 (Protein Data Bank code: 7ZLH). *C*, thrombospondin repeat domains have two regular β-strands and one rippled strand, which contains the WxxWxxWxxC sequence. The three tryptophans on the A strand can be *C*-mannosylated and are intercalated by three arginines from the *B* strand, forming the tryptophan–arginine ladder. Three conserved disulfide bonds are labeled in *yellow*. A fucosylation site directly precedes the second conserved cysteine. The figure displays a TSR of properdin (Protein Data Bank code: 6RUS). TSR, thrombospondin type 1 repeat.

An alternative way to distinguish glycosylation reactions is to consider the involved glycosyltransferases. Most proteins first pass through the endoplasmic reticulum (ER) and then through the Golgi to reach their final destination. A typical Golgi glycosyltransferase, either acting directly on proteins or on extending glycans, has a type II membrane orientation with a short N-terminal cytoplasmic tail, one transmembrane domain, and a luminal catalytic domain (2). These enzymes use nucleotide-linked monosaccharides as activated donor substrates. Glycosyltransferases acting in the ER either utilize nucleotide-activated sugars or lipid-linked donor sugars as a substrate. The *C*-mannosyltransferases, together with the *O*-mannosyltransferases, the oligosaccharyltransferase, and the glycosyltransferases that built up the lipid-linked oligosaccharide or the glycosylphosphatidylinositol anchor, form the glycosyltransferase-C (GT-C) superfamily (3). All these enzymes are proteins with multiple transmembrane domains (11–14), with little overall homology to each other. Still, a common membrane topology and conserved, putative, catalytic residues can be identified in the whole GT-C superfamily (4). The catalytic subunit of the oligosaccharyltransferase, the *O*-mannosyltransferases, and the *C*-mannosyltransferases thus form an evolutionarily related enzyme family, despite the different generated protein linkages.

## The discovery of *C*-mannosylation

The first report of a hexose modification on tryptophan appeared in 1992 (5). Mass spectrometric (MS) analysis of a neuropeptide of the stick insect *Carausius morosus* showed the presence of a mass corresponding to one hexose linked to tryptophan. The authors could not resolve the exact structure and speculated that the hexose was linked to the indole ring nitrogen of the amino acid. Shortly after, glycosylation of a specific tryptophan in human RNase2 was described by Jan Hofsteenge (6), who can be called the pioneer of *C*-mannosylation, in collaboration with the laboratory of Johannes Vliegenthart. Edman degradation sequencing of the protein N terminus could not confirm the presence of a tryptophan at position 7 that was predicted to be there based on the RNA sequence. This suggested a modification of the amino acid at this position. Initial peptide MS analysis revealed a mass addition of 162 Da to the tryptophan residue that could be confirmed to be a hexose linked to the C2 of the tryptophan indole ring by structural analysis using NMR spectroscopy. Subsequently, the researchers established that the hexose was an α-linked mannose (7) (Fig. 1*B*). Much later, additional analysis confirmed that the initially found modification of the stick insect neuropeptide was a *C*-linked α-mannose as well (8). The existence of an insect protein with *N*-linked tryptophan–mannose has also been reported (9), but no direct structural evidence for this claim has been provided in this publication or afterward.

Around the time of the discovery of protein *C*-mannosylation, Horiuchi *et al*. (10) described a compound with a positive ion mass of 367 Da to be present in human urine. Based on absorption spectra and NMR data, the authors proposed that it was a tryptophan conjugate and named it tetrahydropentoxyline. Later studies, however, established that the 367 Da urine compound was free *C*-mannosyl tryptophan (*C*-Man-Trp) with an identical structure as protein-linked *C*-mannose (11). Interestingly, in the same study, *C*- and *N*-linked conjugates between hexoses and tryptophan could be generated by a chemical reaction under extreme conditions (pH 1 and 80 °C). In addition, tryptophan *N*-glycosides were discovered in food products, and the authors suggested that these were generated by specific enzymatic biosynthesis in fruits (12). All following experiments, however, established a C-C linkage.

*C*-Man-Trp in urine is anticipated to be a degradation product of protein *C*-mannosylation (13) and is under investigation as a marker for kidney function (14) and myelofibrosis progression (15). Glycosylated tryptophan products from fruit are more likely a direct product of enzymatic or chemical reactions with free tryptophan (12). If *C*-linked mannose is eliminated in conjugated form or enzymatically cleaved from proteins or free *C*-Man-Trp is currently unclear. At least, enzymes that allow growth of bacteria on *C*-Man-Trp as the only carbon source exist (16). The exact mechanism of degradation is not known.

## Occurrence of *C*-mannosylation

After the discovery of *C*-Man-Trp in RNase2, *C*-mannose has been reported in many other mammalian proteins (17, 18, 19, 20, 21, 22, 23, 24, 25, 26, 27, 28, 29, 30, 31), in insects (11), mussels (32), apicomplexan parasites (33, 34, 35), and Ebola virus (36, 37) using MS analysis. *C*-mannose has also been observed in crystal structures of mammalian proteins (38, 39, 40, 41, 42, 43, 44) and of *Toxoplasma* (45). Based on these data and the occurrence of *C*-mannosyltransferases (see below), *C*-mannosylation seems restricted to metazoans and alveolates.

Krieg *et al*. (46) determined that in RNase2 the recognition sequence in the protein is WxxW, of which the first tryptophan is *C*-mannosylated. The reaction is enzyme catalyzed (47), and *in vitro*, a synthetic peptide with the sequence WAKW can function as an acceptor (17). Moreover, Krieg *et al*. (46) showed that low *C*-mannosylation was observed with a phenylalanine at position +3. Later, *C*-mannosylation of all three tryptophans in a sequence WxxWxxWxxC has been reported (18, 19). From these observations, the *C*-mannosylation consensus site was described as WxxW/C (Fig. 1*B*). Based on this consensus, Julenius (48) predicted that more than 2000 human proteins could be *C*-mannosylated.

Predicted and confirmed *C*-mannosylation sites have been found in a variety of unrelated proteins. Two families of proteins, however, contain highly conserved sites. The first is formed by the type I cytokine receptors. Most of these membrane-bound proteins contain the WSXWS sequence in a structurally conserved environment, and the presence of this sequence is even used to define type I cytokine receptors (49). *C*-mannosylation of this site was initially confirmed in the erythropoietin receptor by MS (21) and later in the thrombopoietin and granulocyte colony-stimulating factor receptors (27, 50). The crystal structure of the interleukin (IL)-21 receptor also revealed the presence of a *C*-mannose (40). Recently, *C*-mannosylation of the IL-11 receptor was confirmed by MS (51). Based on the conservation of the WSXWS sequence, it can be expected that all type I cytokine receptors are *C*-mannosylated.

A second conserved *C*-mannosylation consensus site is found in thrombospondin type 1 repeats (TSRs) (Fig. 1*C*). TSRs are small and specifically folded elements of 50 to 60 amino acids that occur in a variety of different proteins. Although over 60 distinct human proteins contain 1 to 25 TSRs (52), the roles of these repeats have only been addressed to a very limited degree. TSRs are generally considered to be binding modules (53), for example, to heparin (54). *C*-mannosylated TSRs are abundant on proteins of the complement system or the complement regulator properdin (18, 19). Proteases of the A Disintegrin And Metalloproteinase with ThromboSpondin motif (ADAMTS) family also contain a TSR between the disintegrin-like and the cysteine-rich domains (55, 56). Depending on the actual family member, ADAMTS can have up to 14 additional TSRs in their so-called ancillary domain (55).

The 50 to 60 amino acids of TSRs have a three-stranded fold with three conserved disulfide bridges (Fig. 1*C*) (57). Most TSRs carry at least one, but more often two or three, regularly spaced tryptophan residues upstream of the cysteine, marking the end of the A strand. In the most extended form, a WxxWxxWxxC sequence is present. The tryptophan residues play an essential role in the structure of TSRs, as they associate with the side chains of conserved arginine residues of the B strand (57, 58). This structural motif is referred to as a tryptophan–arginine ladder or simply tryptophan ladder (Fig. 1*C*) (59). Whereas *O*-fucosylation of serine and threonine has been shown to be required for secretion of at least some TSRs (60, 61, 62), the function of *C*-Man is largely unknown. Intriguingly, the WSXWS motif of the IL-11R is also part of a tryptophan–arginine ladder, a structural motif that appears to be crucial for proper folding of the respective domain of the cytokine receptor (63, 64). C-mannosylation is, however, not an exclusive feature of thrombospondin type 1 repeats or type I cytokine receptors and can be found in other proteins containing a WXXW motif, such as the myelin-associated glycoprotein, glucosidase II, or the chaperone DNAJC3 (42, 65).

## The *C*-mannosyltransferases

The first *C*-mannosyltransferase was identified from the *dpy-19* mutant of the nematode *Caenorhabditis elegans* (66, 67). The DPY-19 protein displays similarities with the catalytic subunit of the oligosaccharyltransferase and other glycosyltransferases of the GT-C superfamily, which suggested that it used a dolichol-phosphate substrate. Experiments demonstrated that DPY-19 modifies the WxxW motif of the TSR-containing proteins MIG-21 and UNC-5 and that the absence of *C*-mannosylation affects the cellular expression level of these proteins (66).

Unlike *C*. *elegans*, which expresses a single *C*-mannosyltransferase, mammals exhibit four paralogs, named DPY19L1, DPY19L2, DPY19L3, and DPY19L4. In humans, expression of DPY19L1, DPY19L3, and DPY19L4 is ubiquitous, whereas DPY19L2 is restricted to the testis (68). The genes *DPY19L1* and *DPY19L3* are present in all vertebrates and have originated from gene duplications that occurred before the divergence of the fish lineage. An additional duplication prior to mammalian divergence generated *DPY19L2* (69). The paralogs exhibit different specificities for acceptors. Using CRISPR–Cas-engineered Chinese hamster ovary (CHO) cells, Shcherbakova *et al*. (70) demonstrated that DPY19L1 and DPY19L3 were sufficient for full *C*-mannosylation of the mouse netrin receptor UNC5A. DPY19L1 was able to modify the first two tryptophan residues of the WxxWxxWxxC motif, whereas DPY19L3 acted on the third. To date, the proteins modified by DPY19L2 and DPY19L4 are unknown.

The nature of amino acids surrounding the tryptophan residue influences the process of *C*-mannosylation. The *C*-mannosyltransferases require an amino acid with a small side chain at position +1 but are tolerant to structure variability at position +2 (48, 71). Their action is also hindered by the presence of a cysteine residue at position −2, explaining the absence of *C*-mannose in the mucin Cys domain despite the presence of a WxxW motif (72). Mutagenesis experiments revealed that substituting the conserved cysteine at position −2 of the WxxW motif enabled *C*-mannosylation of the Cys domain. In agreement, this observation, introducing a cysteine at −2 position in the homologous Cys domain of human cartilage intermediate layer protein 1, prevented *C*-mannosylation of this protein (72).

Recently, the cryogenic-electron microscopy structures of *C*. *elegans* DPY-19 provided a structural basis of acceptor sequon and donor substrate binding and enabled the proposition of a reaction mechanism for *C*-mannosylation (71). To bind to the *C*-mannosyltransferase, the acceptor sequon has to bend sharply next to Trp(0), which is incompatible with any secondary protein structure, confirming that, like *N*-glycosylation, *C*-mannosylation occurs before protein folding. The Leu474 residue of *C*. *elegans* DPY-19 and its equivalent residues in the mammalian paralogs dictates the sequon specificity. This residue is conserved in DPY19L1, whereas DPY19L3 and DPY19L4 exhibit a tyrosine that would clash with the indole of W(+3) and prevent *C*-mannosylation of the WxxW sequon. In DPY19L2, a methionine is present at this position. Other residues that form the acceptor recognition site and the presumed catalytic base are conserved in human DPY19L2 but are partly missing in DPY19L4. To date, the *C*-mannosyltransferase activity of these two paralogs has not been experimentally demonstrated. However, deleterious mutations in the DPY19L2 gene, including mutation of the acceptor-binding site, are associated with globozoospermia and cause male infertility (73, 74). It should be underlined that *C*-mannosylation of several proteins lacking the consensus WxxW or WxxC motifs has been reported (65, 75). It remains to be established if these are substrates of DPY19L4.

**Figure 2:**
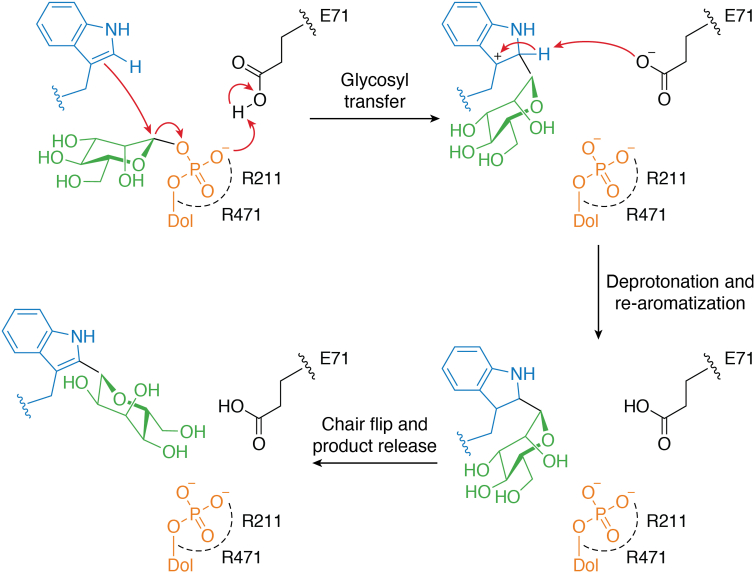
**Proposed catalytic mechanism for *C*-mannosyltransferases** (adapted from Ref. (71) licensed under a Creative Commons Attribution 4.0 International License http://creativecommons.org/licenses/by/4.0/).

*C*-mannosyltransferases are inverting enzymes that specifically use dolichyl phosphate mannose as a donor substrate and follow an electrophilic aromatic substitution mechanism (Fig. 1) (71). Conformational changes occurring upon acceptor peptide binding are required for the donor substrate to adopt an active conformation. In this conformation, the anomeric C1 carbon of the mannose is positioned in close proximity to the C2 carbon of the acceptor tryptophan. The catalytic base, likely represented by Glu71 in *C*. *elegans* DPY-19, may abstract a proton from the C2 of the tryptophan indole group for catalysis to proceed. Interactions with Arg211 and Arg471 stabilize the negative charge of the departing dolichyl phosphate–leaving group and facilitate the electrophilic attack of the indole on the anomeric carbon of mannose. The catalytic base subsequently regenerates the indole aromaticity *via* deprotonation. Finally, the geometry of the active site suggests that the immediate product has a ^4^C_1_ chair conformation (Fig. 1). A conformational flip of the mannose from ^4^C_1_ to ^1^C_4_ may hence accompany product release from the enzyme (Fig. 2).

## Conformation of *C*-mannose

**Figure 3:**
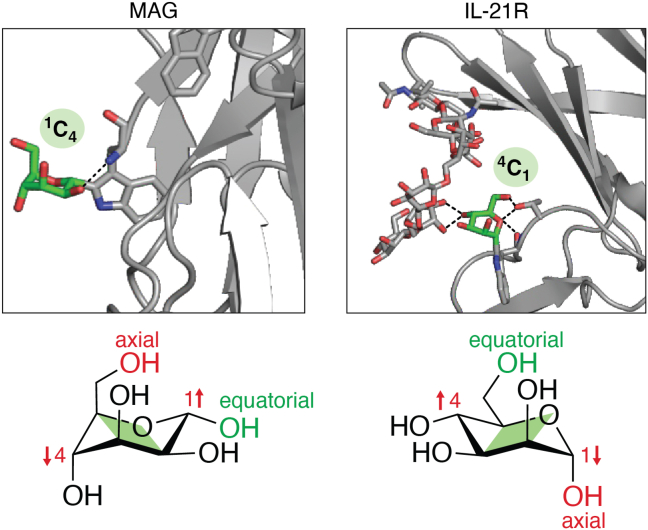
**Chair conformation of *C*-mannoses in proteins**. In the majority of *C*-mannosylated proteins, such as the myelin-associated protein (MAG, Protein Data Bank code: 5LFR), the α-mannose preferably adopts a ^1^C_4_ conformation. In contrast, the *C*-mannose on W195 of the interleukin 21 receptor (IL-21R, Protein Data Bank code: 3TGX) adopts a ^4^C_1_ chair conformation. Hydrogen bonding of the 4-OH of the *C*-mannose with the *N*-glycan substituting N54 restricts movement of the α-mannose and forces it into the unfavored ^4^C_1_ chair conformation. Additional intramolecular hydrogen bonds indicated by *black dashes* stabilize the *C*-mannose conformation.

Carbohydrates, as monomers or linked, can take different conformations (Fig. 3). Although other forms can exist, for aldohexoses in the pyranose form, chair conformations are considered the stable forms. Two main factors determine which of the two possible chair conformations, ^1^C_4_ or ^4^C_1_, is the dominant form. Ring substituents that are equatorial are considered energetically favorable, whereas the anomeric effect between the ring oxygen and the C1-linked oxygen, when present, favors an axial orientation of the C1 substituent. Robert Woods (76) explained the finesses of this in an excellent review about carbohydrate modeling. In β-d-glucose, all substituents are equatorial in the ^4^C_1_ chair conformation, making this conformation by far the most stable form. Also α-d-glucose, with the C1 substituent now axial, and the C2 and C4 epimers, mannose and galactose, are in any structural analysis found, or assumed, to be preferably in the ^4^C_1_ chair conformation. This can be explained by the fact that the substituent formed by C6 is in all cases equatorial. This is clearly illustrated by the fact that if β-glucuronic acid (in ^4^C_1_) in heparin is converted by glucuronyl C5-epimerase, the formed iduronic acid preferably takes a ^1^C_4_ conformation in which C6 is equatorial, but all other substituents are now axial (77). A similar situation occurs when α-mannose is not *O*-linked but *C*-linked. A tryptophan carbon is linked to the mannose C1, which is axial in the ^4^C_1_ conformation and equatorial in the ^1^C_4_ conformation. *C*-linked α-mannose thus has two carbon atoms as ring substituents to C1 and C5, of which one is always equatorial and the other axial in either ^4^C_1_ or ^1^C_4_ conformation. In addition, the anomeric effect around C1 is no longer present. In the original publication in which *C*-mannose was first described, Hofsteenge *et al*. could not ascertain a dominant conformation of *C*-mannose in the RNase2-derived peptide and ascribed this to the opposing influence of the C5-linked hydroxymethyl group and the indole ring of tryptophan linked to C1 on the conformation of *C*-mannose.

Later conformational analyses by NMR of free *C*-Man-Trp, either isolated from natural sources (11, 78) or synthesized (79, 80), proposed a nonexclusive preference for the ^1^C_4_ conformation in the free amino acid form. Interestingly, Nishikawa *et al*. (80) also synthesized α-*C*-glucose linked to tryptophan, which rather adopted a ^4^C_1_ conformation. Glucose, the C2 epimer of mannose, has three equatorial OH groups (at C2, C3, and C4) in this conformation, which obviously pushes the equilibrium toward the ^4^C_1_ conformation.

What about the conformation in proteins? Löffler *et al*. (81) analyzed the conformation of *C*-Man-Trp in intact RNase2 by NMR and compared this with a peptide derived from the same protein. They concluded that *C*-mannose takes a different conformation in the protein, in which mannose is rotated around the Trp C2 and Man C1 bond, but that the tryptophan linked to C1 remains in an equatorial position with the mannose in either ^1^C_4_ or a skew boat conformation.

Only more recent X-ray crystallography studies allowed observing *C*-linked mannoses in a fixed position in protein structures. In a structure of the IL-21 receptor, the electron density allowed *C*-mannose to fit only in a ^4^C_1_ conformation (40), whereas in different structures of thrombospondin repeat and Ig domain–containing proteins, the electron density rather permitted the presence of *C*-mannose in the ^1^C_4_ conformation (42, 43, 44, 45) (Fig. 1). The difference is that in the IL-21 receptor, the mannose movement seems restricted by interaction with an *N*-linked glycan linked to another domain of the IL-21 receptor, whereas in a thrombospondin repeat, the mannose seems to be able to freely adopt its preferred ^1^C_4_ conformation. Molecular dynamic simulations of an intact thrombospondin repeat did not reveal a preference for either the ^1^C_4_ or ^4^C_1_ conformation. Both conformations presented hydrogen interactions with the surrounding amino acids and maintained the tryptophan–arginine ladder (82).

All data are in accordance with a preference of *C*-Man-Trp for the ^1^C_4_ conformation, which is supported by theoretical considerations. The preference for one conformation, however, seems not as high as for *O*-linked monosaccharides. It can be forced into the ^4^C_1_ conformation in the IL-21 receptor, and *C*-mannose has also been reported to bind to the lectin *Galanthus nivalis* agglutinin that is only able to bind to α-mannose in the ^4^C_1_ conformation (22). The latter property is, however, not the exclusive privilege of *C*-mannose. An α-mannosidase involved in *N*-glycan processing forces the mannose in the ^1^C_4_ conformation in the initial step of the hydrolysis (83).

## Function of *C*-mannosylation

Although numerous *C*-mannosylated proteins have been identified, there is even today little evidence of the actual function of this post-translational modification. Answering this question represents an experimental challenge: it is rather easy to mutate the tryptophan residue to which the mannose is usually attached and to determine whether this mutation has a functional consequence for the protein. However, it is not possible from such an approach to determine whether the observed effect stems from the loss of the mannose alone or rather from the loss of the tryptophan residue and resulting destabilization of the tryptophan ladder.

Nevertheless, numerous studies made use of such a mutational approach. Sasazawa *et al*. (27) analyzed *C*-mannosylation of the thrombopoietin receptor, which belongs to the type I cytokine receptor family. They identified five tryptophan residues that are *C*-mannosylated and created three different receptor variants in which one or two tryptophan residues were replaced by phenylalanines. They not only found impaired activation of downstream signaling cascades for the two double mutants but also significantly reduced cell-surface amounts of the mutated receptors, making it difficult to judge whether the impaired signaling was really because of the lack of mannosylation or rather a consequence of the altered amount of receptor at the cell surface (27). *C*-mannosylation was confirmed for ADAMTS-like 1/Punctin-1 at Trp39 and Trp42, which are part of a typical WxxW motif (25). Here, the authors could show that mutation of the tryptophan residues to alanine or phenylalanine reduced protein secretion, which was also the case when they expressed Punctin-1 in CHO-Lec35.1 cells, a *C*-mannosylation–defective cell line (25, 84).

The importance of the WSXWS motif for the biological function of type I cytokine receptors was shown nearly 3 decades ago, for example, for the erythropoietin receptor (85). Mutagenesis studies of the IL-11R confirmed that mutations of the tryptophan residues resulted in reduced amounts of the IL-11R at the cell surface (63). The mutated receptor variants were retained within the ER and could not proceed further through the secretory pathway, most probably because of misfolding of the respective domain (63). Recently, the mutation Trp307 to Arg307 of the IL-11R, which affects the second tryptophan residue of the WSXWS motif, was shown to result in the complete absence of the IL-11R from the cell surface and in two homozygous individuals in craniosynostosis because of the lack of IL-11 signaling (64). However, these effects probably result from the absence of the tryptophan residue, and whether the lack of the mannose residue alone would have the same or a different effect on the IL-11R has not been investigated so far. Due to the recently determined structure of the IL-11 signaling complex, it is quite clear that neither the WSXWS motif of the IL-11R nor the WSXWS motif of the signal-transducing β-receptor gp130 participates in binding of the ligand IL-11. Interestingly, structural data of IL-21 in complex with the IL-21R revealed not only that the first tryptophan of the WSXWS motif is *C*-mannosylated but also that this mannosylation forms a hydrogen bonding network with the *N*-linked glycan that is attached to Asn54 of the IL-21 (Fig. 3) (40). This study represents a rare example of an actual function of the attached mannose residue. It would be intriguing to express the IL-21R in a cell line that is incapable of *C*-mannosylation and to compare the structure and biological function of such an IL-21R variant to unequivocally show the function of the *C*-mannosylation. Whether such linkage to an *N*-glycan can also be found in other type I cytokine receptors or whether this is a unique feature of the IL-21R has not been investigated so far.

As mentioned above, such mutagenesis approaches limit the ability to get significant insight into the actual importance of *C*-mannosylation. However, using cells deficient in *C*-mannosyltransferase(s) allowed the detailed analysis of several substrate proteins. A defect in secretion and cell surface expression of the netrin receptor UNC5 occurred when the protein was expressed in CHO cells lacking DPY19L1 but not DPY19L3 (70). In contrast, knockout of *DPY19L3* in the human fibrosarcoma cell line HT1080 was shown to decrease the formation of vasculogenic mimicry and proliferation, suggesting the importance of *C*-mannosylation in cancer (86). Significantly, Shcherbakova *et al*. (82) demonstrated that the presence of *C*-mannoses increases the resistance of TSRs to thermal and reductive denaturation and promotes oxidative folding *in vitro*. Molecular dynamics simulations suggested that the *C*-mannose orients the tryptophan residue, hereby facilitating the formation of the tryptophan–arginine ladder and disulfide bridge formation.

**Figure 4:**
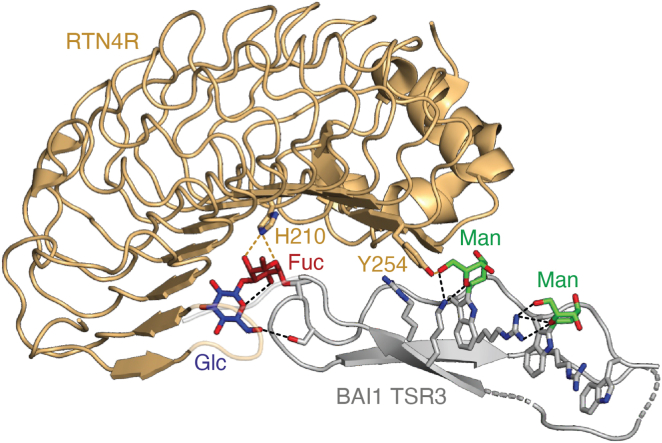
**Binding of the brain-specific angiogenesis inhibitor 1 (BAI1) to the reticulon-4 receptor (RTN4R) involves *C*- and *O*-glycans**. The structure of BAI1 TSR3 (in *gray*) bound to RTN4R (in *light orange*) (Protein Data Bank code: 7R84) highlights the interaction of the *C*-mannose O6 hydroxyl on Trp418 with Tyr254, as well as the binding of the *O*-fucose on BAI1 Thr424 to His210. Additional intramolecular hydrogen bonds are shown in *black dashes*.

Hydrogen bonding of *C*-mannose with arginine and other charged or polar amino acids has been shown to stabilize *C*-mannosylated proteins (87, 88) and promote protein–protein interaction (89). Brain-specific angiogenesis inhibitors (BAIs) are adhesion-G-protein–coupled receptors whose extracellular sequences contain four or five *C*-mannosylated TSRs. The transinteraction of BAIs with reticulon-4 receptors (RTN4Rs) at the surface of neurons or glia cells plays a role in dendritic arborization and synapse formation. The high-resolution structure of BAI1 TSR3 with RTN4R revealed that besides stabilizing the TSR *via* intramolecular hydrogen bonds, the *C*-mannose O6 hydroxyl on Trp418 forms a hydrogen bond with Tyr254 of RTN4R (Fig. 4). The complex also involved hydrogen bonding between the *O*-fucose on BAI1 Thr424 and His210 of RTN4R. Mutations of His210 and Tyr254 in RTN4R abolished complex formation, demonstrating the crucial role of the glycans in the protein–protein interface (89).

The role of *C*-mannosylation has also been investigated in several organisms. In the nematode *C*. *elegans*, deficiency of DPY-19 results in the polarization and left–right asymmetrical migration of Q neuroblast (66, 67). This phenotype is more penetrant at higher temperatures and can be linked to a defect in the MIG-21 protein, which regulates the sensitivity of Q neuroblasts to the Wnt/β-catenin signaling pathway (67, 90). The absence of *C*-mannosyltransferase is expected to affect the cellular level and secretion of additional proteins, such as UNC-5, which would explain the lethal phenotype observed at temperatures above 24 °C (66, 67). The function of *C*-mannosylation in neuronal migration seems to be evolutionarily conserved. Mouse DPY19L1 has been shown to be highly expressed in glutamatergic neurons and is required for their radial migration during corticogenesis (91).

In medaka fish, no major impairment of the early embryonic development was observed upon deletion of the gene encoding DPY19L3 (*dpy19l3*). Nevertheless, a small percentage of the *dpy19l3*^−/−^ fishes presented an incomplete closure of the optic fissure that could be associated with a reduction of ADAMTS16 secretion (92). In contrast, deletion of *dpy19l1* in medaka fish led to a shortening of the anterior–posterior axis and caudal bending (92). Severe developmental defects were also observed in zebrafish deficient in *dpy19l1* (93). The observed severe curvature of the body axis was associated with a lack of *C*-mannosylation of the subcommissural organ-spondin protein. Subcommissural organ-spondin was expressed and secreted but failed to assemble to form the Reissner fibers, suggesting an impact of *C*-mannosylation on the protein interaction (93).

The importance of *C*-mannosylation has also been studied in the apicomplexan parasites *Toxoplasma gondii* (34), *Plasmodium berghei* (94) and *Plasmodium falciparum* (95, 96). These parasites express a single *C*-mannosyltransferase able to fully *C*-mannosylate the sequence WxxWxxC (97) and several TSR-containing proteins important for various parasite processes and life cycle stages. Deletion of the DPY19-encoding gene in *T*. *gondii* impaired adhesion of the parasites to host cells, with severe consequences on motility and host cell invasion. This phenotype was connected to a marked cellular decrease of the micromenal protein MIC2, likely because of protein misfolding and degradation by the ER-associated degradation. In addition, the lack of *C*-mannosyltransferase led to a strong attenuation of virulence and induced protective immunity in mice that cannot be explained by the observed MIC2 decrease. Hence, the loss of *C*-mannosyltransferase activity impacts additional proteins that have not yet been identified (34).

Malaria parasites express several members of the TRAP (thrombospondin-related anonymous protein) adhesin family required for transmission to mosquitoes. The absence of *C*-mannosylation particularly compromised the stability and protein expression levels of MTRAP (merozoite TRAP-like protein) and the secretion of CTRP (circumsporozoite and TRAP-related protein) from the ER (96). Consequently, gametogenesis and ookinete infection of the mosquito midgut were impacted, and the parasite transmission was blocked (94, 96). In contrast, deficiency of *C*-mannosyltransferase had no impact on the asexual stages of *P*. *falciparum* and *P*. *berghei* (94, 95, 96). Altogether, studies in parasites show that *C*-mannosylation stabilizes TSR proteins needed for the life cycle.

## Conclusion and outlook

The initial discovery that a tryptophan residue within a protein can be modified by the addition of a hexose was more than 3 decades ago. Nevertheless, we still know comparatively little about the molecular mechanisms behind this fascinating post-translational modification and even less about the impact of *C*-mannosylation on the function of the individual proteins.

The crucial importance of *C*-mannosylation on the level of the whole organism is well established. Global deficiency in *C*-mannosylation, usually achieved through genetic knockout of one of the transferases, has a broad impact, as seen through developmental defects in zebra and medaka fish. Knockout of the *C*-mannosyltransferase in the parasite *T*. *gondii* reduced its ability to adhere to and thus invade its host cells, and knockout of *DPY19* in *P*. *falciparum* blocked its transmission because of interference with its life cycle. In humans, so far only mutations in the *DPY19L2* gene are known, which are associated with globozoospermia and cause male infertility. Importantly, the *C*-mannosylated proteins that are targets of DPY19L2 are not yet known. Human individuals with mutations in the other three genes encoding the *C*-mannosyltransferases have not been reported yet, but one would expect an even stronger phenotype, which could resemble the congenital disorders of glycosylation seen in patients with defective *N*-glycosylation (98).

The identification of the WxxW motif led to the discovery of thousands of putative substrates for *C*-mannosylation. Nevertheless, for only a few of them, *C*-mannosylation has actually been shown to take place, and most of the crystal structures of proteins with a WxxW motif lack the *C*-mannose residue. The unavailability of easy-to-use tools to study *C*-mannosylation might contribute to the small number of studies in this regard, as MS with manual curation of the spectra is still the most common way to determine whether a protein is *C*-mannosylated or not. Antibodies or lectins that selectively recognize *C*-mannosylated proteins, as well as algorithms for the automated annotation of mass spectra, would facilitate their identification. The cryogenic-electron microscopy structure and catalytic mechanism of *C*. *elegans* DPY-19 have been elucidated and provide insight into the specificity of *C*-mannosyltransferases, but the enzymatic activity and specificity of DPY19L2 and L4 still have to be established. Defining the fine specificity of the enzymes would enable us to predict more accurately the extent of *C*-mannosylation. Similarly, we still know considerably little about the function of *C*-mannosylation on the level of the individual protein. The most clearly established biological function appears to be that *C*-mannosylation assists in folding of the protein and increases its structural stability. Consequently, the absence of *C*-mannosylation destabilizes the proteins, which results in their retention and degradation in the ER. However, how strong these effects are and how much the fate of the individual proteins depends on *C*-mannosylation of a single tryptophan residue varies from protein to protein. In some instances, the *C*-mannose can also play a direct role in protein–protein interactions. Whether these are the only functions of this particular post-translational modification has still to be determined.

## Conflict of interest

The authors declare that they have no conflicts of interest with the contents of this article.
